# Perceptions, attitudes and training needs of primary healthcare professionals in identifying and managing frailty: a qualitative study

**DOI:** 10.1007/s41999-020-00420-0

**Published:** 2020-10-30

**Authors:** Christina Avgerinou, Marina Kotsani, Magda Gavana, Martha Andreou, Dimitra-Iosifina Papageorgiou, Violeta Roka, Despoina Symintiridou, Chrysanthi Manolaki, George Soulis, Emmanouil Smyrnakis

**Affiliations:** 1 Department of Primary Care and Population Health, University College London, Rowland Hill Street, London, NW3 2PF UK; 2 Université de Lorraine, CHRU-Nancy, Pôle “Maladies du Vieillissement, Gérontologie et Soins Palliatifs”, 54000 Nancy, France; 3 Laboratory of Primary Health Care, General Practice and Health Services Research, Aristotle University of Thessaloniki, Thessaloniki, Greece; 4 Avdira Health Center, Abdera, Xanthi Greece; 5 Farkadona Health Center, Farkadona, Trikala Greece; 6 1st TOMY Serron, Serres, Greece; 7 Rodolivos Health Center, Rodolivos, Serres Greece; 8 Outpatient Geriatric Assessment Unit, Henry Dunant Hospital Center, Athens, Greece

**Keywords:** Frailty, Geriatrics, Primary care, Education, Health professionals, Qualitative research

## Abstract

**Purpose:**

Although frailty can be delayed or prevented by appropriate interventions, these are often not available in countries lacking formal education and infrastructure in geriatrics. The aim of this study was to: (a) explore ideas, perceptions and attitudes of primary health care (PHC) professionals towards frailty in a country where geriatrics is not recognised as a specialty; (b) explore PHC professionals’ training needs in frailty; and (c) define components of a frailty educational programme in PHC.

**Methods:**

Qualitative design, using two focus groups with PHC professionals conducted in Thessaloniki, Greece. Focus groups were audio recorded and transcribed. Data were analysed with thematic analysis.

**Results:**

In total 31 PHC professionals (mean age: 46 years; gender distribution: 27 females, 4 males) participated in the study (physicians *n* = 17; nurses *n* = 12; health visitors *n* = 2). Four main themes were identified: (1) Perceptions and understanding of frailty; (2) Facilitators and barriers to frailty identification and management; (3) Motivation to participate in a frailty training programme; (4) Education and training. The main barriers for the identification and management of frailty were associated with the healthcare system, including duration of appointments, a focus on prescribing, and problems with staffing of allied health professionals, but also a lack of education. Training opportunities were scarce and entirely based on personal incentive. Professionals were receptive to training either face-to-face or online. A focus on learning practical skills was key.

**Conclusion:**

Education and training of professionals and interdisciplinary collaboration are essential and much needed for the delivery of person-centred care for people with frailty living in the community.

**Electronic supplementary material:**

The online version of this article (10.1007/s41999-020-00420-0) contains supplementary material, which is available to authorized users.

## Key summary points

**Aim:**

To explore the perceptions and attitudes of primary health care (PHC) professionals towards frailty in a country where geriatrics is not recognised as a specialty, and to explore their training needs in the identification and management of frailty.

**Findings:**

The main barriers towards identifying and managing frailty are associated with the healthcare system, with the most important ones identified to be a gap in geriatric education and training of professionals, as well as problems with staffing of allied health professionals (AHPs) in community settings. However, PHC professionals are motivated and receptive to training in frailty, and they particularly value interactive learning with a focus on practical skills.

**Message:**

There is an imperative need for education and training of PHC professionals, recruitment and training of AHPs and interdisciplinary collaboration for the delivery of person-centred care for people with frailty living in the community.

**Electronic supplementary material:**

The online version of this article (10.1007/s41999-020-00420-0) contains supplementary material, which is available to authorized users.

## Introduction

Frailty is a common age-related, complex and multidimensional clinical entity. Frailty is very common in later life, with prevalence rates varying across different studies and settings. It is estimated to affect around 11% [1] of community-dwelling people aged ≥ 65 in developed countries, and 17% in low- and middle-income countries [2]. Frailty is characterised by an accelerated failure of homeostatic mechanisms, triggered by a stressor, of even minor intensity, leading to disproportionate decline and adverse outcomes [3]. It is associated with an increased risk of hospital admission [4], falls [5], delirium [6], disability [7], transition to long term care [8] and death [9]. However, frailty can often be reversed or attenuated by implementing appropriate interventions [10–12]. The notions of Integrated Care for Older PEople (ICOPE) and intrinsic capacity have been introduced by WHO to attribute positive features and emphasise the role of asset-based interventions in the prevention and management of frailty [13, 14].

Although such interventions are increasingly incorporated in healthcare systems of many countries, this is not the case for countries who lack the resources or the capacity. In Greece in particular, as well as in some other Balkan countries [15], geriatric medicine does not exist as a medical specialty. Moreover, general practice is a relatively new specialty, and the organisation and provision of primary care services are characterised by inconsistencies in delivery across different regions, which has been further delayed due to adverse economic circumstances [16]. With a population that continues to age, Greece has one of the highest percentages of people above the age of 80 (6.5%) in Europe [17]. The Survey of Health, Aging and Retirement in Europe (SHARE) identified a prevalence of frailty 14.7% and prefrailty 44.9% amongst 939 people aged ≥ 65 in Greece [18]. In view of population ageing and the current economic situation, Greek primary care physicians are the first (and often the only) port of call for the older population.

However, and especially considering the lack of formal geriatric education, clinicians may not be equipped with the skills required to deal with the complicated needs of older people living with frailty. Training primary health care (PHC) professionals in how to identify and manage frailty are essential given the scope of the problem. The philosophy of family medicine includes a person-centred approach that takes into account individual goals of care, beliefs, preferences, social context, and, as such, it can expand on these core values and skills when caring for frail older people [19].

The aim of the present study was to (a) explore the ideas, perceptions, and attitudes of PHC professionals towards frailty in a country where specialist geriatric care is not established; (b) explore PHC professionals’ training needs in frailty; and (c) define the main components of a training programme aiming to support health professionals at identifying and managing frailty in primary care.

## Methods

### Design

The study design is qualitative using focus groups with health professionals. This study is nested within a mixed-methods research project with the overarching objective to plan, implement and assess the feasibility and efficacy of an educational program aiming at training PHC professionals in the identification, assessment and management of frailty. The study protocol was presented at the 15th International Congress of the European Geriatric Medicine Society (EuGMS) [20].

The session took place face-to-face on a single day. An introductory 10-min presentation was given by the principal investigator in the lecture theatre at the start of the session to welcome the participants and explain the objectives and timetable of the day. After some brief introductions, participants were divided into two breakout rooms where focus groups were held. Focus groups were followed by a training session on frailty delivered to all participants, led by geriatricians-experts in the field. Questionnaires were administered to participants before the seminar, upon completion on the day, and 3 months later. In this article, we report the specific methods and results that apply to the qualitative part of the project (focus groups), whereas those of the quantitative part (questionnaires) will be presented in another publication.

### Setting

Two focus groups with PHC professionals took place in November 2019, at premises of the Aristotle University (AUTH) in Thessaloniki, Greece. The study population consisted of PHC physicians, nurses and health visitors who were employees of public sector primary care services in a catchment area covering parts of Macedonia, Thrace and Thessaly in Northern Greece. There were representatives from both rural and urban PHC settings.

### Recruitment

Recruitment was facilitated by the AUTH Primary Health Care Research Network (AUTH.PHC.RN). Professionals were invited by e-mail to take part in the study, selected by the following inclusion criteria: (a) General Practitioners (GPs) or General Internal Medicine (GIM) specialists working in the community (collectively referred to as Primary Care Physicians (PCP) onwards), nurses and health visitors, who work in PHC and deal with older people in everyday clinical practice); (b) interested in taking part on a voluntary basis (no remuneration provided); (c) being available to physically attend a 1-day research and training event on a particular date; (d) given written informed consent to participate. There was also an expectation that they would participate in the programme paired with a PHC professional employed by their respective PHC service (e.g. physician and nurse, or physician and health visitor) so that they could collaborate in applying knowledge into clinical practice following completion of the training.

### Data collection

In view of study design and available resources, a convenience sample was used, and the overall number of participants was limited to those who could attend on that day. A topic guide was developed prior to the study and reviewed by the research team (ESM Appendix 1). It consisted of questions around the main topics of exploring attitudes towards frailty, perceptions of PHC professionals’ training needs regarding the identification and management of frailty, and what are the main features of a training programme, including learning objectives, format, and frequency. Two focus groups with PHC professionals were conducted in parallel and each focus group was co-facilitated by two researchers, with an additional researcher who kept field notes. Focus groups were audio recorded with consent, transcribed verbatim and identifiable data was anonymised.

### Data analysis

Transcripts were read by all members of the research team. Thematic analysis [21] was used to identify key emergent themes and their meaning. The analysis team (all authors) identified a preliminary thematic framework. A coding framework was developed, agreed and applied to all transcripts. NVivo software (version 11, QSR International) was used to facilitate data management. The coding framework was applied to the transcripts and refined iteratively. The overall interpretation of meaning and implications for practice were considered by the research team, who brought expertise in general practice, geriatrics, frailty, medical education and qualitative research methods. Selected illustrative quotes (translated from Greek to English) are presented.

### Ethics approval and consent to participate

The study protocol received ethical approval by the Medical School Bioethics Committee of the Aristotle University of Thessaloniki. All participants signed informed consent prior to their inclusion in the study. Withhold of consent for recording precluded participation in the focus groups, but not to the rest of the seminar.

## Results

A total of 31 PHC professionals participated in the study. The median age was 46 years. A summary of characteristics is presented in Table 1.

**Table 1:** Participants’ characteristics (*n* = 31)

Characteristic	*N*	%
Gender		
Male	4	12.9
Female	27	87.1
Age (years)		
Missing	6	19.4
≤ 39	6	19.4
40–49	9	29.0
50–59	8	25.8
> 60	2	6.5
Profession		
PCP (GP)	14	45.2
PCP (GIM specialist working in the community)	3	9.7
Nurse	12	38.7
Health visitor	2	6.5
Professional experience (years)		
< 5	4	12.9
5–10	7	22.6
11–15	8	25.8
16–20	6	19.4
> 20	6	19.4

Four main themes emerged from the analysis of the focus groups: (a) perceptions and understanding of frailty; (b) facilitators and barriers to frailty identification and management; (c) motivations to participate in a training programme; (d) education and training in frailty.

### Perceptions and understanding of frailty

#### Perception of frailty as physiological ageing and multimorbidity

Various views on the meaning of frailty were expressed during the focus groups. It is worth noting that the majority of participants had limited prior knowledge of frailty as a term. Hence, the discussion evolved based on ideas and perceptions that were formed through clinical experience. For many of the participants, frailty was a synonym to ageing and was closely related to the biological age of the individual, the wear and tear of the body, as well as the presence and accumulation of chronic health conditions.

Among the comorbidities mentioned were hypertension, heart disease, diabetes, osteoporosis, falls, and cognitive impairment. There appeared to be some confusion between frailty and disability in the sense that the association between the two notions was not clear. Disability was often perceived as a sign of frailty rather than an outcome of frailty.

#### Perception of frailty as vulnerability and the role of stressors

The perception of frailty as vulnerability or sensitivity was proposed by many participants, although different ideas came to light about its meaning. For some this vulnerability had more of a psychological or emotional dimension. There was a spectrum of views regarding the type and degree of psychological vulnerability, ranging from loneliness leading to attention-seeking behaviour, psychosomatic problems, but also bereavement and overt depression.

For others frailty was more organic or physical in nature. Features reported to be indicative of frailty included: slowness, mobility issues, cognitive impairment, functional decline, change from a previous state, frequent attendance and increased use of health services.

The role of stressor events in the manifestation of frailty was thoroughly discussed by participants. Health professionals perceived frailty both as an underlying factor which increased vulnerability to stress as well as the consequence of a cascade of stressors.

For some participants, the most important stressor was psychological stress, often as a result of an important life event, such as the death of a spouse or child, move to a new environment or other home changes. Moreover, physical conditions e.g. fever or an infection were thought to play an important role in triggering functional decline.

Socioeconomic stressors were also highlighted, with examples reported of older people facing financial difficulties or social isolation.

Selected quotes are presented in Table 2.

**Table 2:** Theme 1: perceptions and understanding of frailty

Subtheme	Illustrative quote
1.1 Perception of frailty as physiological ageing and multimorbidity	
Understanding of frailty as ageing and the accumulation of long-term health conditions	“I am talking about frailty. And he [the patient] is carrying with him the diseases he had since he was young, since he was younger… It can be atherosclerosis, it can be heart disease, an operation, a bypass. And all this causes a wear, meaning that as he becomes older, he becomes even frailer, as years pass by and he ages further. And this is what I bring to my mind as frailty”. (Focus group 2)
Disability perceived as a sign of frailty	“Yes, [frailty is] a decline of [the patient’s] functions, first regarding how he can look after himself, how he responds to his needs at home, as well as more generally in the society”. (Focus group 2)
1.2. Perception of frailty as vulnerability and the role of stressors	
Frailty perceived as psychological vulnerability	“I understand it [frailty] differently. I understand it as that a patient - is not a patient. He is simply an aged person who thinks he has some problems, whereas essentially, that means organically, he does not.” (Focus group 1)
Physical frailty (e.g. slowness, memory issues, muscular wasting)	“It is the patient who will take a lot of time to sit down, to take out his medication booklet (it might even fall off his hands). He may have forgotten a couple of medicines, may have forgotten that medication has been prescribed already, and we talk about the same things over and over again, and when he goes away, I will be frustrated because it will take him a long time to do so, because he is extremely slow. Or even with some muscular wasting, relatively skinny, not that confident, robust person, who would leave [practice premises] easily. This is how I imagine someone who comes in with frailty.” (Focus group 1)
Inability to cope as a result of being exposed to a stressor (e.g. emotional stress)	“And you see that [the older person] is, say, depressed, you see that [he/she] cannot cope with everyday routine, and taking the history you see that the person has ended up in this state after a stressor. Whereas previously, with the same routine, and medication, [he/she] was coping. Eventually the stressor has made [him/her] frail and [he/she] cannot cope anymore.” (Focus group 1)
Physical stress (e.g. infection)	“I feel that frailty in older people is a greater vulnerability which makes them react more ‘intensely’ (intense can be many different things) to a stressor. For example, an older person who also has frailty may react very differently to an infection. And this can drift the whole system.” (Focus group 2)
Socioeconomic stress	“The psychological, the social background certainly plays a role, but the physical as well; I think that the physiological age-related wear of the body plays a big part, and with the slightest trigger [the person] is more vulnerable than the others who are in better physical state; I think that nutrition plays a big role, older people do not usually have a nutritious diet, they cannot cook, they cannot afford anymore to feed themselves properly, and this shows from their blood tests; beyond all, they also have an instability, meaning their muscular system is affected.” (Focus group 1)

### Facilitators and barriers to frailty identification and management

Among the factors reported to facilitate or hamper the identification and management of frailty were factors related to the healthcare system, the patient, and the healthcare professional. Selected quotes are presented in Table 3.

**Table 3:** Theme 2: facilitators and barriers to frailty identification and management

Subtheme	Illustrative quote
2.1 Factors related to the healthcare system	
Duration of appointment	“PCP(1): And to take a good history requires time. OK, sometimes in our practices we may not have the required time to understand ourselves what exactly is wrong with our patient. PCP(2): Fifteen minutes is the time you have to examine. PCP(1): Yes, but these fifteen minutes are… PCP(3): Not to examine. To finish, so that the patient is gone. Out of the door. PCP(1): Yes, but it is what you said. From the moment he comes in, to open his bag, to tell you ‘Doctor, what did I forget, yes, all this. I don’t know if this is all done in those fifteen minutes” (Focus group 1)
Disempowerment of professionals	“There is more emphasis on prescribing, that’s what I see in reality, and everything else is underrated. There is no reason to have a health visitor, a social worker, a physiotherapist, these disciplines are considered redundant. What they offer is not necessary, that’s how this is viewed, that’s what I realise.” (Focus group 1)
Staffing of AHPs and availability of MDT	“I would like to say that the team is a basic condition for [managing] frailty, because I also think that this concerns more the other health professionals than the doctors… I believe that a doctor can make a diagnosis that this patient is frail, but the management falls more on the shoulders of other health professionals. The interventions of the doctor will be minimal, when there is a new illness, because I think that interventions that need to take place in frailty are more relevant to the nursing care and management, or even education of the family or carers about how to look after a frail patient.” (Focus group 2)
2.2 Factors related to the patient	
Fear of stigmatisation	“On the basis of not accepting vulnerability, or frailty, [the older person] may develop a particularly demanding behaviour towards health professionals. It is what we call ‘difficult patient’. A form of dealing with a difficult patient, and it can create tension” (Focus group 2)
2.3 Factors related to healthcare professionals	
Culture of cooperation with a team	“PCP(I): I myself have not learned to cooperate with a team. This, I think, is the most basic. We have not learned, particularly we physicians, have not learned, we have never encountered it, when and how to cooperate. PCP(II): Yes. PCP(I): … we do not know exactly which roles they [allied professionals] have and how we could cooperate for a better result. Each and every one takes isolated actions, I think.” (Focus group 1)

#### Factors related to the healthcare system

One of the main problems associated with the structure and organisation of the healthcare system was the lack of adequate consultation time, as well as the focus of the existing PHC system on repeat prescriptions. It was reported that in most urban practices the scheduled length of appointment is 15 min, which was deemed to be inadequate for full history taking and examination, especially when the patient is older and has slowness, multiple complaints or complicated history. The geographical location of practices was thought to have an impact on appointment length and availability as well as home visits, the latter depending on availability of transport, distance from the nearest hospital, or dominant mentality of those involved.

Another common problem was the disempowerment of PCPs, resulting from a misconception that they should be focused towards prescribing and administration. Patients often tended to consult different doctors, many of them private specialists, which led to a fragmented approach to medical care as opposed to the continuity of care. This culture was reported to be associated with higher risks of inappropriate prescribing and iatrogenic problems and examples were illustrated.

Disempowerment of other disciplines was also thought to be at the heart of problems associated with staffing in PHC. Participants reported a shortage of allied health professionals (AHPs) (physiotherapists, occupational therapists, dieticians, social workers, psychologists, etc.) due to funding issues, but also because their role was deemed more important in hospital. As a result, there was no multidisciplinary team (MDT) available in the community in most cases.

The lack of MDT was reported to be one of the main barriers to responding to the needs of frail older people. However, in cases where team members collaborated harmoniously, teamwork was acknowledged as an asset to improving care for older people. Some professionals suggested that in a system with a wider, interdisciplinary team, delegation of tasks could facilitate better time management and a more holistic approach to frailty.

#### Factors related to the patient

Patient-related factors which may play a role in the identification and management of frailty include socioeconomic factors such as social isolation, a language barrier, different culture (e.g. patients belonging in religious or cultural minorities), as well as the role of the family. It was reported that the presence of an accompanying person (usually a family member) was a facilitator when the patient had cognitive impairment, but also a barrier when relatives projected their own ideas, concerns and expectations on the patient, thus making the doctor-patient interaction more challenging.

Another barrier to dealing with frailty was felt to be prejudice on behalf of the older person, fear of acknowledging an increased level of need and refusal of help and support, as ageing and associated dependency were perceived as stigmatising.

#### Factors related to healthcare professionals

The main barriers to identifying and managing frailty in PHC were the lack of education and shortage of AHPs who could form a frailty MDT. Most participants felt they were not equipped with the required knowledge and skills to perform a frailty assessment based on validated tools. Nonetheless, many of them agreed that, even if they had received the necessary education, their work would have been impeded by co-existing practical issues, such as: previously established views and attitudes on behalf of other professionals; the lack of AHPs; resistance to change of established habits; and a lack of motivation, including financial incentives, for the delivery of care which might be perceived as optional or not entirely within the remit of PCPs. The absence of a culture of cooperation and precise knowledge of repartition of roles were also discussed.

Communication among healthcare professionals working in different community services was considered vital. The importance of adequate handover between primary and secondary care was highlighted, as for example upon discharge from hospital where the PCP should be informed by the hospital on whether the patient would need continuing care at home, and this information was reported to be very often missing.

There was an agreement that health professionals working in PHC are in a privileged position due to their previous knowledge of the patient. Some participants thought that personal interest and years of clinical experience combined with their ‘gut feeling’ could help the PHC professional understand the patient better and diagnose/treat the problem, and might, to some extent, counteract the lack in education. However, there was no consensus on this, as some professionals thought that communication skills and doctor-patient relationship were the most important, whereas others prioritised the need for education.

### Motivation to participate in a frailty training programme

There were various motives that led PHC professionals to take part in this training programme on frailty. First, personal motives were discussed, including curiosity about getting to know a new term, research or academic interest and the acquisition or updating of scientific knowledge. A few stated a special interest in understanding frailty in specific vulnerable populations, such as people with dementia and their carers, older refugees, etc. A few others were motivated by the personal experience of being family carers of older people.

Second, other motives were associated with the wider healthcare professional community, such as improving patient care and responding to the needs of their catchment area in view of the prolonged life expectancy and population ageing. Some participants said that their interest was focused on learning frailty screening tools and management strategies, aiming to obtain practical skills which would have direct clinical application. The possibility of reversing frailty in certain instances triggered an interest. A desire to pass knowledge on to other colleagues in the longer term was also reported. Increased awareness was thought to be a motivating factor towards the improvement of the quality of care provided and the relationship with older people.

Third, motives associated with health policies were the search for an alternative model of PHC in combination with the optimal utilisation of scientific human resources and the promotion of interdisciplinary collaboration in the community. Frailty was perceived as a public health issue, given the demographic changes in recent decades. Finally, social perspectives were discussed, such as isolation of older people exacerbated by migration of the younger generations, as well as challenges related to changes in retirement age and the associated expectation that people would be fit in older age and productive for longer.

Selected quotes are presented in Table 4.

**Table 4:** Theme 3: motivation to participate in a frailty training programme

Subtheme	Illustrative quote
Personal motives (e.g. curiosity, academic interest, acquisition of knowledge and skills)	“I am [PCP] and came here today out of curiosity. I am not familiar with the term, although we deal with an older population who comes to the practice. I came to find out what it is and how it could help me in everyday clinical practice, and, moreover the fact… that there is a possibility to reverse frailty, and how this can be achieved.” (Focus group 2)
Motives associated with the wider healthcare professional community (e.g. improving patient care, responding to the needs of the population)	“I am [PCP], dealing with the urban population. I have some relative experience, but I want to organise in my mind the tools for the detection of frailty in this age group and how they can be evaluated and implemented in everyday practice… I am interested in this practical part. I learn about them [the tools], and I see what I do and how I can apply them. This is why I am here.” (Focus group 2)
Increased awareness resulting in better care	“…The more informed you become, the more sensible you become and the more you want to work and get things done and help in what comes up to your daily practice regarding older people.” (Focus group 2)
Motives associated with health policies	“… Frailty starts to become a public health issue. And since we all wish to change even the pension system, we should go back… We should therefore focus on the prevention of frailty of these individuals… Most of them live on their own, they are not nursed by their children anymore. Society changes, everything changes, the pace of life. We are here for this change, so that we can change ourselves for the better. To become equipped.” (Focus group 2)

### Education and training in frailty

#### Previous or current education

There was an agreement among all participants that there is a major gap in education regarding the care of older people. The vast majority of participants had not received any training in geriatrics, and more specifically in frailty, as part of their undergraduate or postgraduate education. Care of older people did not officially form part of specialty training in GP or GIM, and geriatric medicine has not yet been recognised as a medical specialty or subspecialty in Greece. Two postgraduate programmes relevant to ageing were reported to be more recently available at a national level. Few participants had attended a conference as part of which there was a round table or other session with an ageing topic.

Overall, training opportunities were felt to be scarce and entirely based on personal incentive and a self-motivated search for continuing medical education rather than forming part of a standard curriculum. An additional barrier to accessing geriatric educational resources was mentioned to be the lack of material and clinical tools available in the native language.

#### Training which is considered desirable or needed

Participants reported various objectives of a training programme depending on their previous experience with the topic, their expectations and motivations. A large group of PHC professionals aimed for learning and using validated tools and practical techniques for the management of frailty. There was a clear preference for knowledge that could be directly put into practice. Frailty prevention and reversion were among the desired learning outcomes described.

Training in communication skills when dealing with older people was considered an important component of an educational programme because a gap in communication skills training was perceived to be a common issue among newly qualified doctors and nurses by those who were more senior.

Preferred methods that were reported regarding education format included online training (either on an individual basis or in small groups), the opportunity for a placement in geriatric care units, and practical skills demonstration. This could be provided with either face-to-face or to a small group of learners in a local practice, with peer learning and supervision by a trainer, which could be transmitted online via an electronic platform to enable sharing of experiences and interaction with other learners.

There was no concrete view regarding the frequency in which participants would prefer to be trained. Many participants shared reservations that they could not be granted study leave frequently or for a long period of time, therefore, online training was more pragmatic for the majority. Moreover, participants unanimously thought that education should be continuous and not once only and most agreed on the necessity of education in geriatrics as a compulsory part of GP specialty training as well as training for other health professionals looking after older people.

There was an agreement to promote scientific communication, feedback and interaction with colleagues to revise, evaluate and maintain knowledge. The development of an electronic platform in the form of a forum was suggested, to trigger active participation through discussion of cases and problem solving, which should preferably become available via a trustworthy source (e.g. university) and be independent of social media.

Selected quotes are presented in Table 5.

**Table 5:** Theme 4: education and training in frailty

Subtheme	Illustrative quote
4.1 Previous or current education	
Lack of educational resources in native language	“…once again alone I started to read what was this frailty all about…it has been very difficult to find questionnaires in Greek, there were questionnaires in English but it is not appropriate to do the translation myself… because Greek literature on the [frailty] syndrome was very limited…” (Focus group 1)
4.2 Training which is considered desirable or needed	
Preference for knowledge with direct clinical application	“Because from one theoretical seminar to everyday practice, when you are pressurised by other things, there can be a significant gap… and I want to see how many of these can be really applied.” (Focus group 2)
Frequency of training—Peer learning	“It therefore depends on how often you will do it. To my point of view, the shorter the time that does not allow you to forget previous [knowledge], the better. Meaning that if in a semester I could come, say, twice a week, or talk to you somehow, or present what I did, how, did I recognise it correctly, someone else to check me (because to tell the truth we do not know), or discuss ‘I did these interventions in this patient’, and see later if they had a benefit or not, and you tell me ‘yes, and there is also that intervention’, I think that even in 6 months I could maybe do something, and once a year I might discuss with other colleagues, not necessarily with you, I think it would help.” (Focus group 1)

## Discussion

This focus groups study exploring PHC professionals’ views highlights that the main barriers hampering the identification and management of frailty are associated with the healthcare system, including appointment length and availability; a current focus of primary care on prescribing; a model of fragmented care; and problems with staffing of AHPs, making the creation of an MDT impossible in current terms. The main barriers associated with healthcare professionals are the lack of education and difficulties in communication between primary/secondary care and across disciplines. Participants had not received any formal training in geriatrics as part of the undergraduate curriculum or medical specialty training. Training opportunities were scarce and entirely based on personal incentive. Professionals were receptive to different forms of training including face-to-face and online methods, and group practice with peers. A focus on learning practical skills was considered essential.

The main strength of this work is that it is, to the best of our knowledge, the first study exploring the perceptions and attitudes of PHC professionals on frailty that has been conducted in Greece, a country where geriatric medicine does not exist as a specialty/subspecialty, opportunities for geriatric education are scarce and frailty as a term is largely unknown. The participants’ limited previous education and experience on geriatrics and frailty and the conduction of the focus groups before the seminar created a naïve basis for the investigation of perceptions and attitudes, without the bias of previous exposure to relevant information.

We interviewed PHC professionals including physicians, nurses and health visitors from across urban and suburban areas and various locations covering different administrative regions in Northern Greece to capture diversity in views and practices. Furthermore, within the research team there was expertise in general practice, medical education and geriatrics, which allowed a contribution to the interpretation of findings from different angles.

We used a focus groups qualitative methodology to obtain our data, which provided the advantage of including a larger number of participants interviewed collectively and hence more quickly compared to individual interviews. Although this setup prompted discussion and exchange of views to take place among the participants, it might also mean that some personal opinions have been missed or not explored in depth, despite the facilitators’ best effort to encourage active participation of all.

Moreover, the study population consisted of a fair number of PHC professionals, but, due to limited resources, we did not have the capacity to recruit a larger number of participants. It is possible that, if we had the capacity to hold more focus groups, we might have captured additional themes and perspectives. However, this study gives insight into an important under-researched topic and it is the starting point for more work to be done.

A knowledge gap around frailty has been recognised by previous research. A qualitative study exploring the understanding of frailty among European healthcare policy makers reported a lack of awareness among general clinicians and AHPs [22]. Although some policy makers demonstrated an understanding of the malleability of frailty, others implied that frailty was a normal part of ageing and as such it was not preventable or reversible. This resulted in a restricted ownership of frailty by specialists, whereas it was suggested that this should be devolved to a wider healthcare audience [22].

Psychological and socioeconomic factors were perceived to have a major impact on frailty by PHC professionals in our study. Similarly, in another study examining everyday frailty management strategies by Polish stakeholders, professionals stated that frailty was often initiated by a lack of mental wellbeing, due to psychological hardships, social isolation and loneliness, and they suggested that frailty is caused by the psychological inability to cope with illness [23]. In a Swedish study exploring healthcare professionals’ views, frailty was perceived as a multidimensional concept with several interacting aspects. Although the answers that professionals gave on frailty definition differed from the current state of knowledge, they reflected a holistic approach and a comprehensive understanding of the concept. Moreover, in the same study ‘being bodily weak and ill’ and ‘being dependent in everyday life’ were reported as features of frailty by healthcare professionals, whereas activity limitations and dependency are traditionally described by the term of disability, which is a consequence of frailty [24]. This is a similarity with our own study where there appeared to be an overlap between frailty and disability based on some participants’ views.

Regarding the identification of frailty, an Australian study showed that GPs very often use a type of rapid, intuitive screening of older patients, instead of a formal screening tool [25]. A Canadian study explored family physicians’ clinical ‘gestalt’ (‘gut feeling’ or intuition) impressions of their older patients using a think-aloud approach. The study showed that physicians struggled to conceptualise frailty without a formal definition. Factors that they considered before determining a patient’s frailty status included physical characteristics, functional characteristics and living conditions [26].

Communication skills are thought to be important when dealing with older people and a lack of training in this domain is reported in our study. This is in line with other studies which showed that information needs to be perceived as relevant to the older person’s needs and tailored to their situation to promote motivation and engagement with health promotion services for frailty [11, 27].

The need for interdisciplinary training on frailty and frailty tools has been previously highlighted by a UK study exploring the views of community care staff, including healthcare assistants, nurses, occupational therapists, physiotherapists, psychiatric nurses, social workers and therapy assistants. In this study, there was a consensus across all specialties that the assessment of frailty requires a holistic approach. Professionals worked together by performing joint visits, taking part in MDT meetings, making referrals and sharing information via computer records. Community professionals wished to receive more training, ideally face-to-face, in an environment that would facilitate peer learning across a range of specialties. Similar to our findings, participants of the UK study emphasised the need for training with practical benefits to clinical practice as opposed to one that is theoretical [28].

There is an imperative need for training of primary care and other community professionals in identifying, preventing and managing frailty. Apart from continuing professional education, geriatrics should form part of undergraduate education, as well as postgraduate curricula, at least for GP and GIM specialty training. Previous research has shown that undergraduate geriatric medicine teaching improves medical students’ conceptualisation of frailty and their understanding of structured geriatric assessments and management plans [29].

Education should be provided in a format that is accessible and focussed on practical learning with a demonstration of skills, including validated frailty screening and geriatric assessment tools, and training in communication skills. Material in native language could facilitate access to knowledge, thus validated translations are required. Peer learning and continuing education via a trustworthy professional forum or network need to be taken into consideration when designing frailty training programmes for health professionals. Physical attendance and time limitations should also be considered. Group education was welcomed by participants and might increase the uptake and applicability of knowledge.

The development and delivery of education in frailty is a starting point but it is not enough on its own, especially in countries with no established geriatric facilities and referral systems. It should be accompanied by the evolution of primary care to meet the original goal of a holistic, person-centred approach, and the development of integrated care. A shift in culture is required, promoting the empowerment of AHPs through training and recruitment of staff. Interdisciplinary collaboration is key to the successful organisation and implementation of a person-centred model of care for frail older people.

## Conclusions

Primary care professionals could play an important role in the prevention, identification and management of frailty in older people living in the community. However, they often feel overwhelmed by the workload, not confident in dealing with frailty and limited by systemic deficits. This is more prominent in countries such as Greece, where there is no established geriatric medicine training. An important gap in education and training of health professionals in how to identify and manage frailty has been identified in this study.

Despite numerous systemic challenges, PHC professionals are receptive to training via a range of methods including face-to-face or online, provided that interactive peer learning, demonstration and practice of skills are included. Along with the need for education, recruitment and training of AHPs is required to deliver an integrated care model with interdisciplinary collaboration and a holistic approach to frailty.

## Electronic supplementary material

Below is the link to the electronic supplementary material.

Supplementary material 1 (DOCX 15 kb)

## Data Availability

The datasets generated during and/or analysed during the current study are not publicly available due to containing potentially identifiable information but may be available from the corresponding author on reasonable request, and subject to conforming to general data protection regulation (GDPR).

## Abbreviations

AHP: Allied Health Professional
AUTH: Aristotle University of Thessaloniki
AUTH.PHC.RN: Aristotle University of Thessaloniki Primary Health Care Research Network
GIM: General Internal Medicine
GP: General Practitioner/General Practice
MDT: Multi-Disciplinary Team
PCP: Primary Care Physician
PHC: Primary Health Care
SHARE: Survey of Health, Aging and Retirement in Europe
UK: United Kingdom
WHO: World Health Organisation
