# Supplementary material for: Perceptions, attitudes and training needs of primary healthcare professionals in identifying and managing frailty: a qualitative study
Source: Eur Geriatr Med. 2020 Oct 30;12(2):321–32. doi: 10.1007/s41999-020-00420-0 (PMC7990835; doi:10.1007/s41999-020-00420-0)
Supplement: Supplementary file 1 — Supplementary material 1 (DOCX 15 kb) [file 41999_2020_420_MOESM1_ESM.docx]

**Focus groups with primary health care professionals on the identification and management of frailty in older people**

**Provisional topic guide**

**Before the start:**

1. Introductions of facilitators and co-facilitators. Information on the objectives and structure of the session.
2. Written informed consent for participation and audio recording of the session. Information that data will be made anonymous after transcribing and before the analysis, so that individuals cannot be recognised in the transcript.
3. There is no right or wrong answer
4. Information on the procedure so that all views can be heard (e.g. step down when someone has talked enough, to give the opportunity to others to talk as well)
5. Introduction of all participants
6. Questions and clarifications

**Α. General views and perceptions of frailty**

- How do you perceive frailty in older people as health professionals through your clinical experience?
- Which factors can help you recognise that an older person presents features of frailty?
- Which factors can prevent you from recognising signs of frailty?
- How could care of older people with frailty be improved at your place of work / at the region of your practice?

**Β. Views of the training needs of primary health care professionals for the identification and management of frailty**

- Have you received any training on the care of older people? If yes, have you received any training specifically relevant to frailty?
- If yes, what was the format of training (e.g. seminar, conference, face-to-face, online, etc.)?
- What do you think is the content of a training intervention targeting primary health care professionals regarding the identification and management of frailty?
- What skills or knowledge would you like to obtain or improve?
- What format would you prefer for such a training programme?
- What is the frequency and quantity with which you would like to be trained? Within what time frame?
- Individual or group education? Advantages and disadvantages
- How do you think the maintenance of knowledge or skills of health professionals can be assured following the completion of the training programme?

**C. Determination of learning objectives collectively with the team**
